# Knowledge, attitudes, and practices towards urinary system stones among the Chengdu population

**DOI:** 10.1038/s41598-024-60227-y

**Published:** 2024-05-17

**Authors:** Dong Lv, Liangyou Tang, Yongbo Chen, Rui Wang, Ling Liu, Nenghong Jian, Ting Zhang, Taimin Shen

**Affiliations:** 1 Department of Urology, Deyang People’s Hospital, 173 Section One North Taishan Road, Jingyang District, Deyang, 618000 Sichuan China; 2 Department of Health Management and Institute of Health Management, Sichuan Provincial People’s Hospital, School of Medicine,, University of Electronic Science and Technology of China, 32 West Second Section, First Ring Road, Qingyang District, Chengdu, 610072 Sichuan China

**Keywords:** Urinary stones, Health knowledge, attitudes, practice, Health examinations, Lifestyle, Cross-sectional study, Health care, Risk factors, Urology

## Abstract

This cross-sectional study aimed to explore the knowledge, attitudes, and practices (KAP) regarding urinary system stones among the general public in Chengdu, China. Conducted between January and June 2023, this research targeted individuals undergoing physical examinations at the Health Management Center of Sichuan Provincial People's Hospital. Structured questionnaires were administered to collect demographic information and assess KAP related to urinary system stones. Following meticulous scrutiny, 1014 valid questionnaires were retained for analysis. The computed scores for knowledge, attitude, and practice were 9.36 ± 4.23 (possible score range 0–17), 37.75 ± 7.20 (possible score range 11–55), and 30.77 ± 4.00 (possible score range 10–50), respectively. These outcomes suggested insufficient knowledge and moderately positive attitudes and practices among the participants. Structural Equation Modeling (SEM) analysis revealed a direct impact of knowledge on attitude (β = 0.967, P < 0.001), with attitude subsequently exerting a direct influence on practice (β = 0.167, P < 0.001). This indicated an indirect impact of knowledge on practice. Additionally, there was a direct effect of knowledge on practice (β = 0.167, P < 0.001). In conclusion, the general populace in Chengdu exhibited insufficient knowledge and moderate attitudes and practices concerning urinary stones. These findings underscore the imperative for targeted educational interventions aimed at enhancing public awareness and fostering positive attitudes and practices toward urinary stone prevention and management.

## Introduction

The problem of kidney stone disease constitutes a significant global health concern, with notable variations in prevalence rates worldwide. Specifically, these rates range from 1–5% in Asia, 5–9% in Europe, and 7–15% in North America^1^. The disparities are influenced by genetic and dietary factors, resulting in an imbalance in urine composition due to lithogenic substances and inhibitors of crystal formation^2–4^. The occurrence of acute renal colic, resulting from urinary stones, triggers intense pain and obstruction, with potential implications for renal harm and severe complications^5,6^. Urinary stones can manifest anywhere along the route from the kidneys to the urethra, and the severity of pain is more closely associated with the degree of obstruction rather than the size of the stone. Additionally, there exists a possibility of renal calyx rupture and the formation of an urinoma. More concerning is the potential for obstructed renal units to become infected, necessitating urgent surgical drainage due to the ineffectiveness of antibiotics^6^. This disease can impact both children and adults, and it is essential to develop prevention and treatment strategies before the condition exacerbates^7,8^.

Urinary stone development shares overlapping pathways and risk elements with specific cardiovascular conditions, such as coronary heart disease and stroke^9,10^, as well as other health conditions^11,12^. Therefore, patient education plays a crucial role in the early detection of urinary stones. The Knowledge, Attitudes and Practice (KAP) approach has proved to be highly effective in identifying concerns, requirements, and potential obstacles among individuals. This, in turn, contributes to the formulation of educational strategies for disease prevention and diagnosis^13^. In the context of urinary stones, American reports have highlighted a lack of sufficient knowledge among patients regarding the dietary risks associated with nephrolithiasis. However, it is noteworthy that certain individuals demonstrated a willingness to receive dietary guidance as an integral part of kidney stone treatment^14,15^. A further Lebanese study revealed that a significant portion of patients showed keen interest in learning about kidney stone prevention. Moreover, they displayed remarkable adherence when provided with these guidelines in the emergency department^16^.

While stone disease presents a significant health challenge with remarkable onset and recurrence rates, it is essentially a preventable condition. However, there are only a limited number of studies that evaluated the KAP of the general public toward urinary stones^14–16^. Additionally, as far as we are concerned, no such investigation has been conducted in a Chinese population. Thus, this study aimed to explore the KAP of the general public towards urinary system stones in a local healthcare center at Chengdu, China.

## Methods

### Study design and participants

This cross-sectional study was conducted between January to June, 2023, in the Chengdu, Sichuan Province, China, and included general public who attended the Health Management Center of Sichuan Provincial People's Hospital for physical examinations. The inclusion criteria involved individuals aged 18 or older who were willing to participate, while those participants who lacked autonomy were excluded from the investigation. The Medical Ethics Committee of Sichuan Academy of Medical Sciences and Sichuan People's Hospital granted ethical approval for this study, and informed consent was obtained from all participants.

Questionnaires were disseminated to participants by both on-site and online methods to mitigate the risks of selection and non-response biases. The on-site questionnaire was distributed at the Hospital by trained research assistants. Before distribution, these assistants underwent comprehensive internal training to ensure they fully understood the significance and importance of the questionnaires. They also familiarized themselves with the specifications for filling out the questionnaires and were trained in maintaining a polite and friendly manner during interactions with participants. During the collection of the questionnaires, the research assistants diligently checked for any missing items, ensuring the completeness and accuracy of the data collection.

The online questionnaire was conducted through the 'Sojump' platform, with quick response (QR) codes prominently displayed at the medical center. Respondents scanned the QR code to access and complete the survey. In order to maintain data quality and completeness, all questionnaire items were designated as mandatory. The research team undertook a meticulous review of submitted questionnaires, focusing on evaluating completeness, internal consistency, and logical coherence to ensure the robustness of the collected data. This rigorous assessment process aimed to enhance the reliability and validity of the information obtained from the online survey. The on-site questionnaires were also uploaded to the ‘Sojump’ platform to facilitate the generation of a comprehensive dataset for analysis.

### Questionnaires

The questionnaire, designed on the basis of relevant literature references^17,18^, was subsequently modified according to the opinion of 2 senior experts. Redundant or duplicated questions were systematically eliminated, and any ambiguously formulated queries underwent careful refinement to ensure content validity. The final questionnaire was in Chinese and encompassed four dimensions: demographic characteristics (age, gender, body mass index (BMI), residence, education, occupation, environmental temperature at work, monthly income, history of urinary disease, family history of urinary stones, underlying medical conditions), the knowledge dimension, the attitude dimension, and the practice dimension. The knowledge dimension comprised 18 questions, with correct responses to questions 1–14, 16–18 scoring 1 point and incorrect or unclear answers scoring 0 points, yielding a score range of 0–17 points (the correct responses for K2, K6, and K16 were “wrong”). The dimension of attitude comprised 11 questions (where question 2 was reverse-scored), employing a five-point Likert scale. The scale ranged from strongly agreeing (1 point) to strongly disagreeing (5 points), thus yielding a score range of 11 to 55 points. The practice dimension consisted of 10 questions (with reverse scoring for questions 1, 2, 3, and 8), rated from always (5 points) to never (1 point), resulting in a score range of 10–50 points. The questions and the corresponding scores within each dimension enabled the assessment of the KAP levels. Applying Bloom's cutoff method^19^, the knowledge, attitude, or practice was categorized as insufficient, negative, or inappropriate if their score fell below 60% of the total possible score. It was labeled as moderate if the score ranged from 60 to 80% of the total, and deemed sufficient, positive, or appropriate if the score exceeded 80% of the total.

### Statistical analysis

The statistical software employed was the Statistical Package for the Social Sciences (SPSS) 26.0 and AMOS (IBM Corp., Armonk, NY, USA). Continuous variables were presented as mean ± Standard Deviation (SD) and analyzed using one-way Analysis of Variance (ANOVA) or independent samples t-test. Categorical variables were described using frequencies (percentages). Spearman's correlation analysis and structural equation modeling (SEM) were used to examine correlations among the three dimensions. Assumptions of the SEM included: (1) knowledge affects practice; (2) knowledge influences attitude; (3) attitude impacts practice. A significance level of p < 0.05 was considered indicative of statistically significant differences.

### Ethics approval and consent to participate

This work has been carried out in accordance with the Declaration of Helsinki (2000) of the World Medical Association. This work was approved by the Ethics Committee of Sichuan Academy of Medical Sciences and Sichuan People's Hospital (No.2022-449). And informed consent was obtained from all participants.

## Results

A total of 1241 questionnaires were collected. Among them, 46 contained abnormal data, and 182 displayed logical inconsistencies in responses. Following these exclusions, 1014 valid questionnaires were retained. A post hoc reliability analysis was performed, and a Cronbach's α value of 0.862 for formal distribution indicated acceptable internal consistency.

The participants had a mean age of 39.64 ± 12.64 and were mostly females (52.37%), had a body mass index of 18.5–23.9 (54.83%), lived in rural areas (81.46%), had a junior college/bachelor’s degree (51.48%), did not work in the medical field (90.24%), did not frequently work in high temperature environments (91.02%), had a monthly income per capita of 5001–10,000 Chinese Yuan (CNY), did not have relatives with a history of urinary stones (77.32%), had not suffered from urinary diseases before (76.63%), and did not have underlying medical conditions (87.28%). The knowledge, attitude, and practice scores were 9.36 ± 4.23 (on a maximum of 17: 55.05%), 37.75 ± 7.20 (on a maximum of 55: 68.63%), and 30.77 ± 4.00 (on a maximum of 50: 61.54%), respectively, suggesting that participants had insufficient knowledge, moderate attitudes, and moderate practice regarding urinary stones (Table 1).

**Table 1:** Demographic characteristics and KAP scores.

Characteristics	N (%)	Knowledge		Attitude		Practice	
		Score	P	Score	P	Score	P
Total		9.36 ± 4.23		37.75 ± 7.2		30.77 ± 4	
Gender			< 0.001		< 0.001		< 0.001
Male	483 (47.63)	8.39 ± 4.44		36.04 ± 8.04		29.99 ± 3.98	
Female	531 (52.37)	10.25 ± 3.83		39.31 ± 5.93		31.49 ± 3.89	
BMI, kg/m^2^			< 0.001		0.001		< 0.001
< 18.5	63 (6.21)	10.3 ± 3.97		38.7 ± 7.304		30.54 ± 3.868	
18.5–23.9	556 (54.83)	9.92 ± 4.01		38.57 ± 6.573		31.28 ± 4.045	
> 23.9	395 (38.95)	8.42 ± 4.411		36.45 ± 7.823		30.1 ± 3.87	
Residence			< 0.001		< 0.001		< 0.001
Urban	188 (18.54)	7 ± 4.35		34.02 ± 8.22		28.85 ± 3.4	
Rural	826 (81.46)	9.9 ± 4.02		38.61 ± 6.67		31.21 ± 4	
Education			< 0.001		< 0.001		< 0.001
Primary school or below	78 (7.69)	4.64 ± 2.74		30.62 ± 8.33		27.6 ± 3.41	
Middle school/high school/vocational school	201 (19.82)	6.15 ± 3.49		31.04 ± 7.3		28.54 ± 3.19	
Junior college/bachelor's degree	522 (51.48)	10.46 ± 3.81		40.09 ± 5.22		31.44 ± 3.85	
Master's degree or above	213 (21.01)	11.42 ± 3.25		40.98 ± 4.48		32.4 ± 3.76	
Medical related occupation			0.087		0.334		0.023
Yes	99 (9.76)	9.79 ± 4.6		36.84 ± 8.09		29.99 ± 4.45	
No	915 (90.24)	9.31 ± 4.19		37.85 ± 7.09		30.86 ± 3.95	
High environmental temperature at work?			< 0.001		< 0.001		< 0.001
Yes	91 (8.97)	7.18 ± 4.39		33.49 ± 8.87		28.79 ± 4.09	
No	923 (91.03)	9.58 ± 4.16		38.17 ± 6.88		30.97 ± 3.94	
Monthly income, Yuan			< 0.001		< 0.001		< 0.001
< 5000	282 (27.81)	6.93 ± 4.03		33.35 ± 8.07		28.96 ± 3.56	
5001–10,000	418 (41.22)	9.74 ± 4.22		38.44 ± 6.59		31.13 ± 4.02	
10,001–20,000	196 (19.33)	11.07 ± 3.3		40.4 ± 5.07		31.72 ± 3.82	
> 20,000	118 (11.64)	10.98 ± 3.51		41.46 ± 4.58		32.27 ± 3.78	
Family history of urinary system stones			0.001		0.049		0.115
Yes	230 (22.68)	10.23 ± 3.97		38.48 ± 7.22		30.39 ± 4.42	
No	784 (77.32)	9.11 ± 4.28		37.54 ± 7.18		30.89 ± 3.87	
Medical history of urinary system diseases			0.977		0.082		0.416
Yes	237 (23.37)	9.43 ± 4.06		38.54 ± 7.19		30.95 ± 4.05	
No	777 (76.63)	9.34 ± 4.29		37.51 ± 7.19		30.72 ± 3.99	
Underlying medical conditions			0.037		0.831		0.618
No	885 (87.28)	9.45 ± 4.26		37.71 ± 7.22		30.76 ± 4.03	
Yes	129 (12.72)	8.78 ± 4.02		38.06 ± 7.1		30.9 ± 3.79	

In the knowledge dimension, higher knowledge was likely to be found in females (P < 0.001) and those participants who lived in rural areas (P < 0.001), did not frequently work in high temperature environments (P < 0.001), had relatives with a history of urinary stones (P = 0.001), did not have underlying medical conditions (P = 0.037), had lower BMI (P < 0.001), had higher education (P < 0.001), and had higher monthly income (P < 0.001) (Table 1). The answers to the knowledge questionnaire indicated moderate knowledge levels about 11 of all 16 questions. The question with the highest average score of knowledge was *“10. Even after successful treatment of urinary system stones, maintaining a healthy lifestyle and dietary habits is essential to prevent recurrence”* with mean score of 0.74 ± 0.44, and the question with lowest average score was *“16. Cutting back on foods rich in citric acid like oranges, pineapples, and grapes can help in preventing urinary system stones”* with mean score of 0.25 ± 0.43 (Supplementary Table 1).

Better attitude was more frequent in females (P < 0.001) and those respondents who lived in rural areas (P < 0.001), did not frequently work in high temperature environments (P < 0.001), had relatives with a history of urinary stones (P = 0.049), lower BMI (P < 0.001), higher education (P < 0.001), and higher monthly income (P < 0.001) (Table 1). Of a total of 11 questions, the responses in the attitude dimension denoted mostly plain agreement (6 questions), followed by neutrality (3 questions) and disagreement (1 question), together with almost even percentages for neutrality and disagreement in Question 11. The item with the highest average score of attitude was *“9. Ensuring adequate hydration is crucial for preventing urinary system stones”* (3.90 ± 1.13), while *“11. Changing lifestyle and dietary habits to prevent urinary system stones brings about discomfort”* showed the lowest average attitude scores (2.84 ± 1.09) (Supplementary Table 2).

In terms of practice, higher scores were mainly found in females (P < 0.001) and participants who lived in rural areas (P < 0.001), did not work in the medical field (P = 0.023), did not frequently work in high temperature environments (P < 0.001), had normal BMI (P < 0.001), higher education (P < 0.001), and higher monthly income (P < 0.001) (Table 1). The question with the highest average score of practice was *“8. The frequency of delaying urination in my daily life”* (3.55 ± 0.78), with *“occasionally”* being the most frequent answer (44.87%). On other hand, *“1. My level of knowledge concerning urinary system stones is adequate to fulfill my requirements for stone prevention”* had the lowest average score of practice (2.35 ± 1.00), with *“frequently”* as the main answer (38.76%) (Supplementary Table 3).

**Figure 1:**
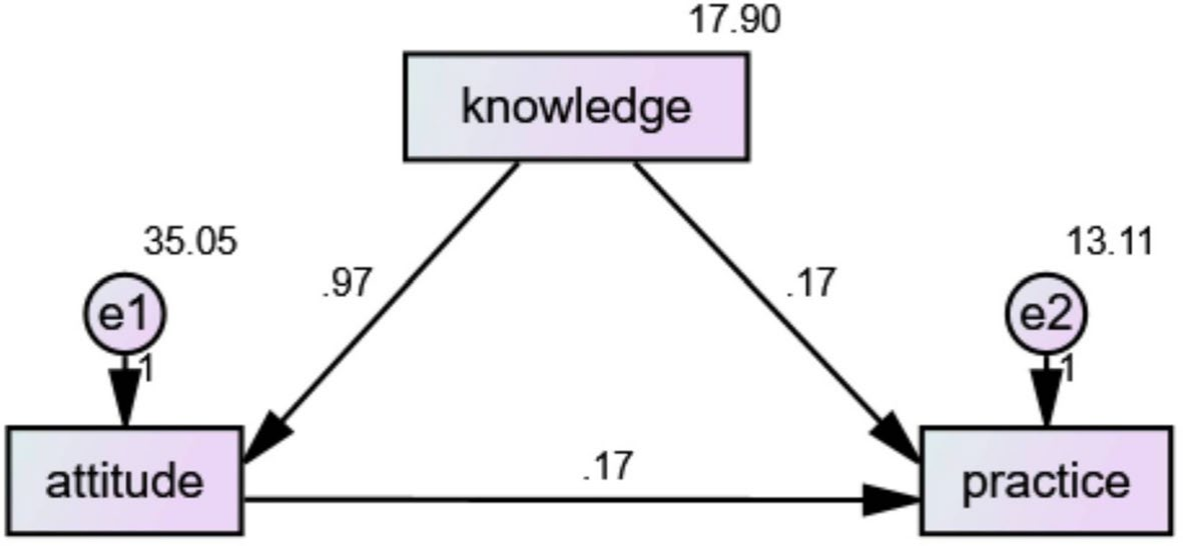
Structural equation modeling.

As shown in Table 2, the Spearman’s correlation analysis showed positive significant correlations between knowledge and attitude (r = 0.555, P < 0.001), knowledge and practice (r = 0.342, P < 0.001), and attitude and practice (r = 0.391, P < 0.001). SEM analysis (Fig. 1) showed that knowledge had a direct positive impact on attitude (β = 0.967; P < 0.001). Additionally, the attitude demonstrated a direct influence on practice (β = 0.167; P < 0.001), indicating an indirect impact of knowledge on practice. Moreover, knowledge also had a direct impact on practice (β = 0.167; P < 0.001) (Table 3). The data fit well into the SEM model, as indicated by RMSEA = 0.075.

**Table 2:** Correlation analysis among knowledge, attitude, and practice.

	Knowledge	Attitude	Practice
Knowledge	–		
Attitude	0.555 (P < 0.001)	–	
Practice	0.342 (P < 0.001)	0.391 (P < 0.001)	–

**Table 3:** Structural equation model.

			Estimate	S.E	C.R	P
Attitude	←	Knowledge	0.967	0.044	21.982	< 0.001
Practice	←	Attitude	0.167	0.019	8.673	< 0.001
Practice	←	Knowledge	0.167	0.033	5.105	< 0.001

## Discussion

The findings of this study shed light on the KAP of the general public towards urinary stones, revealing that the participants exhibited insufficient knowledge, coupled with moderate attitudes and practices concerning urinary stones.. Positive correlations were found between knowledge and attitude, knowledge and practice, as well as attitude and practice. These findings may facilitate the understanding between doctor and patient and foster the creation of innovative educational programs targeting the general population, playing a crucial role in preventing and promptly identifying urinary stones.

The present work identified strong differences in the KAP scores of individuals with different demographic characteristics, including age, gender, BMI, residence, education and other socioeconomic and medical characteristics. A strong association between this disease and socioeconomic status, particularly income, has been previously established^20^, which also reflected on the KAP levels of the patients^21^. Our analysis of respondents revealed a notable deficiency in knowledge concerning urinary stones, particularly in areas related to preventive dietary recommendations and lifestyle habits, such as reducing the consumption of animal protein-rich foods and foods high in citric acid. This deficiency in knowledge was evident in the low scores observed for questions such as *"15. Reducing the consumption of animal protein-rich foods such as meat, eggs, and dairy products can contribute to preventing urinary system stones”* or *“16. Cutting back on foods rich in citric acid like oranges, pineapples, and grapes can help in preventing urinary system stones”*. In this context, there has been limited exploration of the KAP regarding healthy habits and urinary stones among both patients and the general public, both globally and specifically within China^21^. An investigation conducted in Atlanta, United States of America, revealed that local individuals with urinary stones lacked a proper understanding of how to manage their diet and the related risk factors^22^. This emphasizes the crucial need for healthcare providers to take the initiative in explaining and providing guidance on dietary management, with the ultimate aim of improving the comprehension, consciousness, and adherence of patients to these guidelines. In contrast, a study in Beirut, Lebanon, observed that a noteworthy portion of the participants did not receive any recommendations from healthcare professionals in emergency departments upon being diagnosed with urinary stones^16^. As a result, it is imperative for healthcare providers to universally offer succinct health education that takes into account patients' socio-economic backgrounds. This approach is vital as patients place a high priority on their health status^16,22,23^. Both dietary and medical interventions are available for the treatment of urinary stones, with dietary adjustments proving to be effective and cost-efficient. Enhancing awareness about this renal condition can empower patients to recognize its signs, symptoms, and associated risk factors^21^.

Enhancing knowledge has the potential to improve dietary and lifestyle practices, thereby contributing to the prevention of initial occurrences or recurrent episodes^24,25^. This aligns with our findings of an indirect impact of knowledge on practice, as shown by the SEM results. However, in our analysis, we observed that the attitudes of the participants (encompassing beliefs about prevention and the perceived benefits of lifestyle changes) significantly mediated the transformation of knowledge into action. This implies that even with adequate knowledge, the adoption of preventive practices depends heavily on the individual’s attitudinal disposition towards health behaviors. Therefore, the role of attitudes in bridging the gap between knowledge and practice warrants a more detailed discussion, as it is central to the development of more effective health education and intervention strategies. Our results highlight the potential impact of targeted interventions in enhancing awareness and fostering positive practices, particularly among subgroups with distinct demographic characteristics.

Our participants exhibited moderate attitudes and practices towards urinary stones, signaling a need for further enhancement, as seen in the knowledge dimension. For instance, the lowest average attitude score was for the item "Changing lifestyle and dietary habits to prevent urinary system stones brings about discomfort" (score: 2.84 ± 1.09). In terms of practices, the lowest score was for "My level of knowledge concerning urinary system stones is adequate to fulfill my requirements for stone prevention" (score: 2.35 ± 1.00). These responses indicate areas where public perception and behavior might be improved through targeted education and intervention. Comparable results were found in Saudi Arabia, where moderate levels of attitude and practice towards this condition were detected, particularly with more favorable attitudes noted in individuals with higher education and good health status^26^. In a Canadian study of patients who had a history of recurrent kidney stones, less than half of the subjects (45.8%) demonstrated adherence to the best practice guidelines recommended by the Canadian Urological Association^27^. The highest compliance rate (72.6%) was observed in terms of maintaining a high fluid intake, which is considered the most crucial preventive measure against stone formation. Conversely, our respondents showed lower frequency of hydration, with *“sometimes”* being the most common response. In this regard, a notable gap between attitude and practice was observed concerning hydration habits. Although ensuring adequate hydration was recognized as crucial for preventing urinary system stones (high attitude score), there was a lower frequency of actual hydration practice. Another significant gap was observed in attitudes towards dietary and lifestyle changes. The study found that changing lifestyle and dietary habits to prevent urinary stones was seen as uncomfortable or challenging by the participants. This is indicated by the low attitude scores for the related item. While there was an understanding of the importance of lifestyle modifications in stone prevention, the reluctance or perceived difficulty in making these changes points to a barrier in adopting such practices. This gap suggests that merely improving knowledge or positive attitudes may not be sufficient. There is a need to address the practical challenges and psychological barriers that prevent individuals from applying their knowledge and attitudes to their daily habits.

While variations among countries are anticipated due to sociodemographic differences, global health education remains pivotal to ensure accurate adherence to dietary recommendations. Education efforts should be tailored to each patient's socio-demographic circumstances, with particular attention to their level of education. Additionally, achieving an optimal weight by managing BMI is essential, given the significant link between obesity, insulin resistance, and metabolic abnormalities contributing to the rise in urinary stone cases^22,23^. Embracing a healthy lifestyle, encompassing sound dietary habits and regular exercise, emerges as a potent strategy for preventing the recurrence of this medical condition.. Importantly, the present findings could help doctors tailor their communication and education efforts to match the specific background and understanding of each patient, resulting in more effective doctor-patient interactions. This approach can effectively address misconceptions, encourage healthier behaviors, and empower patients, ultimately resulting in improved treatment compliance and better health outcomes.

In light of our findings, it becomes essential to emphasize the dynamic interplay among knowledge, attitudes, and practices in the field of urinary stone prevention. The relationships delineated by the SEM analysis indicate that interventions aimed at urinary stone prevention should not solely focus on knowledge dissemination. Instead, there needs to be a concerted effort to positively influence attitudes, as they play a critical mediating role. For instance, while increasing knowledge about hydration’s role in stone prevention is crucial, fostering a positive attitude towards regular hydration is equally important to ensure that this knowledge is translated into habitual practice. Such an understanding is vital for the design of public health interventions, emphasizing the necessity of not just educating the public but also influencing their attitudes and motivations.

This study has certain limitations that merit careful consideration. Firstly, its single-center design raises concerns about the generalizability of findings to a broader context. While the collected data is valuable, regional factors might exert an influence on the outcomes. Secondly, there is a potential bias towards social desirability in the responses to our survey, wherein participants may be inclined to provide answers that align with perceived societal expectations rather than accurately reflecting their true attitudes or practices. Third, although the sample size is substantial, it may not fully represent the diverse spectrum of knowledge, attitude, and practice levels related to urinary stones. Fourth, we employed two distinct approaches for the study survey, on-site and online, and data from both of the two methods were upload to the ‘Sojump’ platform. Given these circumstances, we are unable to provide specific data regarding biases associated with the collection method. Fifth, besides two independent reviewers, no additional verification of the questionnaire was conducted before formal distribution.

In conclusion, this study offers valuable insights into the KAP of the general public towards urinary stones. The correlations noted between knowledge, attitude, and practice emphasize their interrelation in effective stone prevention. In the future, educational programs tailored for specific regions could enhance knowledge and foster positive attitudes and practices. By addressing demographic differences, especially gender, residence, and socio-economic factors, these interventions could be more impactful. Our findings may serve as a foundation for further investigations in urinary stones that can refine preventive strategies and improve public health outcomes.

### Supplementary Information

Supplementary Tables.

## Data Availability

All data generated or analyzed during this study are included in this published article and its supplementary information files.

## Abbreviations

KAP: Knowledge, attitudes, and practices
SEM: Structural equation modeling
BMI: Body Mass Index
ANOVA: Analysis of variance
SD: Standard deviation
CNY: Chinese Yuan
SPSS: Statistical package for the social sciences
QR: Quick response
