# Supplementary material for: Knowledge, attitudes, and practices towards urinary system stones among the Chengdu population
Source: Sci Rep. 2024 May 17;14:11303. doi: 10.1038/s41598-024-60227-y (PMC11101414; doi:10.1038/s41598-024-60227-y)
Supplement: Supplementary file 1 — Supplementary Tables. [file 41598_2024_60227_MOESM1_ESM.docx]

**Supplementary Table 1.** Distribution of Knowledge Section

| **Knowledge** | **N (%)** | | |
| --- | --- | --- | --- |
|  | Incorrect | Correct | **Average Score** |
| **1. Urinary system stones encompass those found in the kidneys, ureters, bladder, and urethra.** | 309(30.47) | 705(69.53) | 0.70±0.46 |
| **2. Compared to males, females are more susceptible to urinary system stones *.** | 592(58.38) | 422(41.62) | 0.42±0.49 |
| **3. The majority of urinary system stones do not induce typical symptoms before an attack and are often discovered during medical examinations.** | 531(52.37) | 483(47.63) | 0.48±0.50 |
| **4. A family history of stones, metabolic syndrome, kidney diseases, and high-temperature working environments may all elevate the risk of urinary system stones.** | 397(39.15) | 617(60.85) | 0.61±0.49 |
| **5. Urinary system diseases such as urinary obstruction or urinary tract infections can increase the risk of urinary system stones.** | 354(34.91) | 660(65.09) | 0.65±0.48 |
| **6. Consumption of calcium (e.g., taking calcium supplements or regularly consuming calcium-rich foods) within normal limits can lead to urinary system stones *.** | 354(34.91) | 660(65.09) | 0.62±0.49 |
| **7. If left untreated, urinary system stones can potentially impair kidney function and, in severe cases, lead to conditions like uremia.** | 348(34.32) | 666(65.68) | 0.66±0.48 |
| **8. Primary treatments for urinary system stones encompass pharmacologically induced stone expulsion, ultrasonic lithotripsy, and surgical extraction.** | 296(29.19) | 718(70.81) | 0.71±0.46 |
| **9. For smaller stones, adopting lifestyle modifications, increasing fluid consumption, and other non-invasive measures can facilitate natural stone expulsion.** | 312(30.77) | 702(69.23) | 0.69±0.46 |
| **10. Post-successful treatment, it is crucial to sustain healthy lifestyle choices and dietary habits to avert stone recurrence.** | 262(25.84) | 752(74.16) | 0.74±0.44 |
| **11. Diminishing intake of foods high in oxalates, such as black tea, spinach, celery, potatoes, mangoes, and strawberries, is beneficial for stone prevention.** | 478(47.14) | 536(52.86) | 0.53±0.50 |
| **12. Sufficient hydration plays a pivotal role in the prevention of urinary system stones.** | 344(33.93) | 670(66.07) | 0.66±0.47 |
| **13. Restricting foods with high purine content, including organ meats, canned sardines, and various seafood, aids in stone prevention.** | 388(38.26) | 626(61.74) | 0.62±0.49 |
| **14. Regular physical activity and avoiding extended periods of inactivity can contribute to the prevention of urinary system stones.** | 270(26.63) | 744(73.37) | 0.73±0.44 |
| **15. Limiting the intake of animal proteins found in meats, eggs, and dairy products is advantageous in preventing urinary system stones.** | 701(69.13) | 313(30.87) | 0.31±0.46 |
| **16. Reducing consumption of citric acid-rich foods, such as oranges, pineapples, and grapes, supports urinary stone prevention efforts** ***.** | 764(75.35) | 250(24.65) | 0.25±0.43 |

*: The correct answer for these questions was "wrong".

**Supplementary Table 2.** Distribution of the Attitude Section

| **Attitude** | Strongly Agree | Agree | Neutral | Disagree | Strongly Disagree | **Average Score** |
| --- | --- | --- | --- | --- | --- | --- |
| 1. **I believe my current knowledge about urinary system stones suffices for effective stone prevention.** | 118(11.64) | 260(25.64) | 345(34.02) | 252(24.85) | 39(3.85) | 3.16±1.05 |
| 1. **Urinary system stones often manifest with subtle symptoms, thus excessive worry is unnecessary.** | 28(2.76) | 136(13.41) | 283(27.91) | 456(44.97) | 111(10.95) | 3.48±0.95 |
| 1. **Regular screenings for urinary system stones are essential for everyone.** | 137(13.51) | 430(42.41) | 311(30.67) | 123(12.13) | 13(1.28) | 3.55±0.92 |
| 1. **It is vital to maintain a balanced calcium intake to prevent urinary system stones.** | 129(12.72) | 458(45.17) | 321(31.66) | 92(9.07) | 14(1.38) | 3.59±0.87 |
| 1. **Limiting high-purine foods is critical for urinary system stone prevention. Examples include organ meats, canned sardines, and seafood.** | 207(20.41) | 429(42.31) | 249(24.56) | 100(9.86) | 29(2.86) | 3.68±1.00 |
| 1. **It's important to reduce intake of oxalate-rich foods for stone prevention, such as black tea, spinach, celery, potatoes, mangoes, and strawberries.** | 146(14.4) | 348(34.32) | 329(32.45) | 149(14.69) | 42(4.14) | 3.40±1.04 |
| 1. **Decreasing consumption of animal protein-rich foods plays a key role in preventing urinary system stones. This includes meat, eggs, and dairy products.** | 96(9.47) | 267(26.33) | 337(33.23) | 256(25.25) | 58(5.72) | 3.09±1.06 |
| 1. **Cutting back on citric acid-rich foods is beneficial for stone prevention. These include oranges, pineapples, and grapes.** | 108(10.65) | 273(26.92) | 385(37.97) | 188(18.54) | 60(5.92) | 3.18±1.04 |
| 1. **Adequate hydration is imperative for the prevention of urinary system stones.** | 353(34.81) | 398(39.25) | 123(12.13) | 88(8.68) | 52(5.13) | 3.90±1.13 |
| 1. **Participating in moderate physical activity is crucial for urinary stone prevention.** | 361(35.6) | 384(37.87) | 125(12.33) | 88(8.68) | 56(5.52) | 3.89±1.15 |
| 1. **Altering lifestyle and dietary habits to prevent urinary system stones can be challenging.** | 73(7.2) | 212(20.91) | 313(30.87) | 314(30.97) | 102(10.06) | 2.84±1.09 |

**Supplementary Table 3.** Distribution of the Practice Section

| **Practice** | Always | Frequently | Sometimes | Occasionally | Never | **Average Score** |
| --- | --- | --- | --- | --- | --- | --- |
| 1. **I regularly consume animal-derived protein-rich foods, including meat, eggs, and dairy products.** | 214(21.1) | 393(38.76) | 269(26.53) | 117(11.54) | 21(2.07) | 2.35±1.00 |
| 1. **My diet often includes oxalate-rich foods, such as black tea, spinach, celery, and potatoes.** | 77(7.59) | 246(24.26) | 544(53.65) | 127(12.52) | 20(1.97) | 2.77±0.84 |
| 1. **I habitually consume foods high in purines, including organ meats, canned sardines, and a variety of seafood.** | 26(2.56) | 121(11.93) | 437(43.1) | 398(39.25) | 32(3.16) | 3.29±0.81 |
| 1. **I frequently eat foods abundant in citric acid, like oranges, pineapples, and grapes.** | 61(6.02) | 225(22.19) | 485(47.83) | 215(21.2) | 28(2.76) | 3.07±0.88 |
| 1. **I consciously consume over 2500ml of water daily, which equates to roughly 8 to 10 cups or 4 to 5 bottles of mineral water.** | 115(11.34) | 211(20.81) | 356(35.11) | 259(25.54) | 73(7.2) | 3.04±1.10 |
| 1. **I increase my water intake whenever I notice a yellowish tint to my urine or a decrease in urine output.** | 188(18.54) | 350(34.52) | 288(28.4) | 138(13.61) | 50(4.93) | 3.48±1.09 |
| 1. **My hydration schedule is consistent and well-balanced throughout the day.** | 101(9.96) | 229(22.58) | 431(42.5) | 195(19.23) | 58(5.72) | 3.12±1.02 |
| 1. **I typically experience the following frequency of delayed urination in my daily routine:** | 6(0.59) | 72(7.1) | 388(38.26) | 455(44.87) | 93(9.17) | 3.55±0.78 |
| **9. I make certain to engage in sufficient physical activity as part of my routine.** | 58(5.72) | 155(15.29) | 406(40.04) | 310(30.57) | 85(8.38) | 2.79±0.99 |
| **10. I undergo periodic medical check-ups.** | 58(5.72) | 155(15.29) | 406(40.04) | 310(30.57) | 85(8.38) | 3.32±1.29 |
